# Qual é o Cenário Atual da Reabilitação Cardíaca no Brasil e em Portugal?

**DOI:** 10.36660/abc.20220210

**Published:** 2022-05-04

**Authors:** Ricardo Stein, Mauricio Milani, Ana Abreu

**Affiliations:** 1 Universidade Federal do Rio Grande do Sul Porto Alegre RS Brasil Universidade Federal do Rio Grande do Sul, Porto Alegre, RS – Brasil; 2 Fitcordis Medicina do Exercício Brasília DF Brasil Fitcordis Medicina do Exercício, Brasília, DF – Brasil; 3 Hospital Santa Maria CHULN Lisboa Portugal Hospital Santa Maria, CHULN, Lisboa – Portugal; 4 Faculdade Medicina Lisboa Lisboa Portugal Faculdade Medicina Lisboa, Lisboa – Portugal

**Keywords:** Reabilitação, Prevenção Secundária, Exercício, Coração

Brasil e Portugal têm muito em comum e estão ligados por fortes laços de uma história que começou a ser escrita há mais de cinco séculos. A língua é a mesma, embora existam diferenças marcantes no sotaque, assim como no vocabulário. A ponte aérea entre os dois países é intensa (à parte, a pandemia de COVID-19) e cidadãos vindos dos dois lados do Oceano Atlântico habitam ou visitam cidades do além-mar. Enfim, o sangue português corre nas veias (e nas artérias) de muitos brasileiros e, nos últimos anos, uma verdadeira onda de brasileiros imigrou para Portugal, percorrendo o caminho inverso àquele feito por Pedro Álvares Cabral, quando este e suas embarcações chegaram a “Terra Brasilis”.

Agora, talvez confuso, quem lê este texto possa se perguntar: Mas qual a relação das linhas acima com a reabilitação cardíaca (RC)? Qual é o verdadeiro motivo para este texto ser escrito por uma portuguesa e dois brasileiros, todos cardiologistas e adeptos da RC? Na verdade, o objetivo é traçar um breve perfil do que ocorre com a RC nos dois países, tentando informar o leitor a respeito de uma realidade absolutamente desconectada daquela que vemos em outras áreas da Cardiologia. Sem inveja, mas com preocupação enquanto reabilitadores, vemos a Cardiologia florescer em termos de medicamentos e dispositivos que mudam a qualidade e a quantidade de vida dos pacientes,^1^ terapêuticas baseadas nas melhores evidências sendo descritas em Diretrizes, procedimentos invasivos que salvam vidas ou diminuem significativos danos, além de vermos a medicina personalizada “batendo na porta” e prometendo uma potencial revolução no manejo de doenças monogênicas e poligênicas. Enfim, onde há investimento, divulgação e marketing, a Cardiologia é um exemplo inequívoco de sucesso.

É importante salientar que, apesar de ainda se encontrar nos bastidores da terapêutica cardiológica, a RC, em diferentes cenários clínicos,^2 , 3^ é uma estratégia de saúde na qual tanto a classe de recomendação quanto o nível de evidência não deixam nada a desejar, quando comparada com situações auspiciosas como as descritas no parágrafo acima. Revisões sistemáticas e metanálises^4 , 5^ têm mostrado que a sua implementação pode ter um impacto imenso na vida das pessoas, sendo uma ação útil para a sociedade como um todo. Agora, perguntamos: Se um paciente com cardiopatia isquêmica não é antiagregado (seja lá com que fármaco for), o que pensaríamos do cardiologista que cuida desse paciente? Ou se um paciente com dislipidemia com colesterol total de 267 mg/dL e LDL de 191 mg/dL não recebesse uma prescrição de estatina? Ou ainda e “indo mais fundo”, se um paciente com infarto agudo do miocárdio (IAM) com supradesnivelamento de ST não fosse reperfundido, estando disponível um hemodinamista e uma sala de hemodinâmica? Imaginamos que o caso iria para o conselho de ética médica... e com toda razão! No entanto, são poucos aqueles no meio médico que conseguem vislumbrar o enorme vazio que fica, enquanto conduta baseada nas melhores evidências, que é NÃO encaminhar um paciente isquêmico após um IAM ou um paciente com insuficiência cardíaca (IC) de qualquer etiologia para um serviço de RC. Embora, em teoria seja absolutamente entendida a indicação, na prática ela é extremamente subutilizada, seja em Portugal seja no Brasil, como veremos nos parágrafos abaixo.

## Qual é o cenário atual da reabilitação cardíaca no Brasil?

No Brasil o cenário é alarmante, sendo o número de programas de RC documentados extremamente insuficiente para a necessidade clínica, o que certamente é prejudicial para a saúde da população brasileira com doenças cardíacas crônicas. Vamos às evidências: em um estudo publicado em 2020, Britto et al.^6^ estimaram que no Brasil haja apenas uma vaga em um serviço de RC para cada 99 pacientes com cardiopatia isquêmica, sendo esta disponibilidade quase três vezes menor do que a observada em outros 32 países avaliados (uma vaga para cada 32,7 pacientes). Em outro estudo que avaliou a disponibilidade e a densidade mundial da RC,^7^ este tipo de serviço foi identificado em 111/203 países e a relação global de vagas para cada paciente com cardiopatia isquêmica foi de 1:11, o que demonstra uma triste realidade.

Dados não publicados sobre a disponibilidade de serviços de RC^8^ identificaram o funcionamento de apenas 59 programas de RC no Brasil, sendo a maioria destes (71%) concentrados nas regiões Sul (20%) e Sudeste (51%) do país, o que demonstra não só a escassez, como também a heterogeneidade na distribuição nacional. Em relação às vagas disponíveis, a situação fica ainda mais concentrada, visto que 69% delas estão na região Sudeste, o que expõe a grande lacuna de vagas e serviços nas demais regiões brasileiras. A partir dos dados supracitados, os autores também calcularam a disponibilidade nacional de vagas de RC para atendimentos após a alta hospitalar: o número de vagas para atender estes pacientes após o evento cardiovascular representou menos de 2% da necessidade.^8^

Para complicar ainda mais a já crítica situação brasileira, no que diz respeito à pouca disponibilidade de serviços de RC, o início da pandemia COVID-19 impactou seriamente na funcionalidade destes serviços, sendo que 81% dos programas de RC relataram interrupção ou encerramento de suas atividades assistenciais, acrescido de mais 14% de serviços que reduziram o número de vagas até então disponibilizadas.^8^ Não há informações científicas sobre o retorno do funcionamento destes serviços até o presente momento, porém, informalmente, estima-se que a capacidade de atendimento ainda não tenha retornado ao nível pré-pandemia. Além disso, os serviços de RC que reiniciaram suas atividades passaram a receber um novo fluxo de pacientes pós-COVID-19, o que pode ter reduzido ainda mais a disponibilidade de vagas para os pacientes portadores de cardiopatias crônicas ou agudas após alta hospitalar, piorando ainda mais o cenário “catastrófico” da RC no Brasil.

## Qual é o cenário atual da reabilitação cardíaca em Portugal?

Nos últimos anos a RC tem apresentado progressiva melhora, tanto em número quanto em qualidade. No entanto, é sabido que ainda está longe do ideal. Neste cenário, o Grupo de Estudos de Fisiologia de Esforço e Reabilitação Cardíaca da Sociedade Portuguesa de Cardiologia tem realizado questionários recorrentes aos Centros de RC para avaliar a evolução da RC em Portugal.^9 - 13^ No último inquérito, datado de 2019,^13^ foram registrados 25 centros (33% de aumento do número em relação a 2014)^12^ com programas de RC, assim considerados de acordo com padrões nacionais e europeus.^14 , 15^ Destes, 11 centros localizam-se na região norte, 1 na região central, 12 na região da grande Lisboa e 1 no sul do país. Verifica-se que esta concentração de centros diz respeito às grandes cidades e região litorânea do país, o que reflete a grande heterogeneidade com manifesta carência de centros de reabilitação em regiões rurais, nas pequenas cidades e vilas, assim como no interior, o que vai contra às recomendações da Organização Mundial de Saúde para a igualdade no acesso à Saúde.^16^ Se considerarmos que anualmente cerca de 10.000 portugueses têm alta hospitalar por infarto do miocárdio e se estes fossem distribuídos uniformemente no território nacional (o que não é o caso), teoricamente cada centro de reabilitação teria que reabilitar 400 pacientes, só em relação a esta patologia (excluindo-se outras causas de doença cardiovascular). Partindo do princípio que os programas de fase 2 teriam 3 meses de duração, quantos e quais os centros seriam capazes de reabilitar 100 pacientes de cada vez, adicionando aí os números de pacientes com outras patologias? E como conseguir uma fase 3 a longo prazo?

Não é só na distribuição geográfica dos centros em território Português que se verifica essa grande heterogeneidade, mas também nos tipos de programas instituídos, com desenho e duração variável. Em sua maioria, programas de fase 2 apresentam uma frequência semanal de duas a três sessões de exercício (podendo variar de 12 até 36 semanas). Por sua vez, os programas de fase 3 têm uma frequência de uma a três sessões semanais, com opção de prazo de longa duração em alguns casos. Todos os centros têm equipes multidisciplinares, incluindo sempre cardiologista, frequentemente na coordenação dos programas. Além do exercício, os programas incluíram sempre aconselhamento nutricional, a maioria com controle de fatores de risco, alguns com cessação tabágica e com intervenção psicológica. Em outras palavras, isto demanda na preparação de equipes com capacidade para intervir através dos vários componentes da RC, o que já é uma realidade em alguns hospitais e universidades, como acontece com o Mestrado em Reabilitação Cardiovascular da Faculdade de Medicina de Universidade de Lisboa,^17^ curso de formação avançada destinado a diferentes especialistas. Aliás, a oferta de profissionais habilitados pode inclusive ser fator primordial no aparecimento de novos programas de RC.

Outro dado interessante é que em 2019,^13^ verificou-se um aumento de 13% nos pacientes que foram encaminhados para RC, comparativamente ao questionário prévio de 2014,^12^ sobretudo no sistema público de saúde. Entretanto, apesar do aumento numérico de pacientes reabilitados, apenas 9,3% daqueles com alguma síndrome coronariana aguda (SCA – causa mais frequente de indicação de reabilitação), foram de fato reabilitados. Sendo assim, se pensarmos que 100% destes pacientes deveriam participar de programas de RC para melhorar a qualidade de vida e reduzir o reinfarto e a mortalidade,^4 , 18^ estaremos diante de um cenário deveras subótimo. Por sua vez, a IC de diferentes etiologias foi a segunda indicação mais frequente para a fase 2 e 3 (com aumento de 1,8% em relação ao questionário prévio de 2014),^12^ incluindo-se aí a reabilitação após implantação de dispositivos cardíacos (aumento de 3,1%). Seguramente, o encaminhamento para programas de RC em pacientes após SCA ou com diagnóstico de IC necessita ser melhorado, assim como a oferta e a capacitação dos centros.

Causas para não adesão dos pacientes aos programas de RC em Portugal, como em outros países, são a dificuldade de transporte, distância ao centro de reabilitação, problemas financeiros, falta de suporte familiar, falta de motivação, desconhecimento dos benefícios e riscos do exercício, assim como problemas laborais. E para dificultar um pouco mais, nos últimos dois anos, a pandemia COVID-19 veio juntar-se às causas de não adesão, fazendo com que alguns centros desenvolvessem programas à distância para tentar minimizar o obstáculo de isolamento social.

Por fim, apesar de melhorias significativas, a RC em Portugal ainda está longe do que seria o desejável, tanto no encaminhamento, quanto no que diz respeito a uma melhor distribuição de programas pelo país.

## Conclusão

O panorama da RC no Brasil e em Portugal ainda é deficiente e necessita grande investimento, em especial se considerarmos que esta intervenção de prevenção secundária é essencial e pode salvar vidas. Neste contexto e para uma implementação efetiva necessitamos:

1. Promover a divulgação adequada da RC através dos meios de comunicação, assim como estimular políticas de saúde por meio das sociedades científicas e dos próprios profissionais de saúde, tanto a nível individual, quanto institucional;
2. Aumentar o encaminhamento por meio da educação para a prevenção de profissionais de saúde e da criação de um sistema de referenciamento dos pacientes, com indicação tão cedo quanto na alta hospitalar;
3. Combater as causas para não adesão dos pacientes aos programas de RC, tais como: a) dificuldade de transporte e a distância ao centro de reabilitação; b) problemas financeiros e falta de suporte familiar; c) falta de motivação e desconhecimento dos benefícios e riscos do exercício; d) problemas de horário laboral.

Finalmente, é claro para nós que se todos os agentes potencialmente envolvidos no processo de implementação de programas de reabilitação nos dois países (governo, agentes de saúde, sociedade civil, entre outros), investirem “um quinhão”, o resultado irá muito além “do milhão”. Ou seja, ao se fazer uma medicina embasada nas melhores evidências e com um custo relativamente baixo, o resultado pode ser um ganho substancial de vidas, redução de sofrimento e até diminuição significativa em gastos com a saúde da população.
