# Supplementary material for: Qual é o Cenário Atual da Reabilitação Cardíaca no Brasil e em Portugal?
Source: Arq Bras Cardiol. 2022 May 4;118(5):858–60. [Article in Portuguese] doi: 10.36660/abc.20220210 (PMC9368881; doi:10.36660/abc.20220210)
Supplement: Translation

# Translation: What is the Current Scenario of Cardiac Rehabilitation in Brazil and Portugal?

**Authors:** Ricardo Stein, Mauricio Milani, Ana Abreu

**Affiliations:** 1 Brasil Universidade Federal do Rio Grande do Sul, Porto Alegre, RS – Brasil; 2 Brasil Fitcordis Medicina do Exercício, Brasília, DF – Brasil; 3 Portugal Hospital Santa Maria, CHULN, Lisboa – Portugal; 4 Portugal Faculdade Medicina Lisboa, Lisboa – Portugal

**Keywords:** Rehabilitation, Secondary Prevention, Exercise, Heart

Brazil and Portugal have a great deal in common, and they are bound by strong ties, with a history that began to be written over five centuries ago. They share a language, although there are pronounced differences in accent, as well as vocabulary. Air traffic between both countries is intense (much lower during the Covid-19 pandemic), and citizens from both sides of the Atlantic Ocean inhabit or visit cities on the other side. Finally, Portuguese blood runs in the veins (and arteries) of many Brazilians, and, in recent years, a wave of Brazilians have immigrated to Portugal, retracing the path taken by Pedro Álvares Cabral, when he and his 13 vessels first arrived in the land that would receive the name “ *Terra Brasilis* ”.

Confused readers may now be asking themselves, “What is the relationship between the last paragraph and cardiac rehabilitation (CR)? What is the real reason that this text was written by one Portuguese and two Brazilian authors, who are all cardiologists and advocates of CR?” In truth, the objective is to sketch a brief profile of what is taking place with CR in both countries, attempting to inform readers about a reality that is absolutely disconnected from what we see in other areas of cardiology. Speaking not out of envy, but rather out of concern as rehabilitators, we see cardiology flourish in terms of medications and devices that change the quality and quantity of patients’ lives,^1^ therapies based on the best evidence described in the guidelines, and invasive procedures that save lives or significantly reduce damage, in addition to observing that personalized medicine is “knocking on the door” and promising a potential revolution in the management of monogenic and polygenic diseases. In short, wherever there is investment, dissemination, and marketing, cardiology is an unmistakable example of success.

It is important to underscore that, even though it is still behind the scenes in cardiological therapy, CR, in different clinical scenarios,^2 , 3^ is a health strategy for which both the class of recommendation and the level of evidence have left no doubt, when compared to auspicious situations such as those described in the preceding paragraph. Systematic reviews and meta-analyses^4 , 5^ have shown that the implementation of CR can have an immense impact on people’s lives, and it is a useful action for society as a whole. Now, we ask: If a patient with ischemic heart disease does not receive antiplatelet therapy (with any medication whatsoever), what would people think of the cardiologist taking care of this patient? Or what if a patient with dyslipidemia, with total cholesterol of 267 mg/dL and LDL of 191 mg/dL, did not receive a statin prescription? Or, to push things even further, if a patient with an acute myocardial infarction (AMI) with ST elevation did not receive reperfusion therapy, while a hemodynamist and a hemodynamics room were available? The case would imaginably end up before the medical ethics board, and rightfully so! However, few people in the medical field are able to envision the enormous void that remains regarding a conduct based on the best evidence, namely, *not* referring patients who are ischemic after an AMI or patients with heart failure (HF) of any etiology to a CR service. In this case, although the indication is fully understood in theory, it is extremely under-applied in practice, both in Portugal and in Brazil, as we will see in the following paragraphs.

## What is the current scenario of cardiac rehabilitation in Brazil?

The scenario in Brazil is alarming. The number of documented CR programs is extremely insufficient in relation to the clinical need, and this is certainly harmful to the health of the Brazilian population with chronic heart diseases. Let’s look at the evidence: In a study published in 2020, Britto et al.^6^ estimated that, in Brazil, there is only 1 spot in a CR service for every 99 patients with ischemic heart disease, meaning that the availability is almost 3 times lower than that observed in another 32 countries evaluated (1 spot for every 32.7 patients). In another study that evaluated the worldwide availability and density of CR,^7^ this type of service was identified in 111 out of 203 countries, and the global ratio of spots for each patient with ischemic heart disease was 1 to 11, which demonstrates a sad reality.

Unpublished data on the availability of CR^8^ identified the operation of only 59 CR programs in Brazil; most of them (71%) were concentrated in the South (20%) and Southeast (51%) Regions of the country, which demonstrates not only the scarcity, but also the heterogeneity in the national distribution. In relation to available spots, the situation is even more concentrated, since 69% of them are in the Southeast Region, which exposes the large gap in spots and services in other Brazilian regions. Based on these data, the authors also calculated the national availability of CR spots for care after hospital discharge. The number of spots to provide care for these patients after a cardiovascular event represented less than 2% of the need.^8^

To further complicate the already critical situation in Brazil, with regard to the limited availability of CR services, the beginning of the COVID-19 pandemic has had a serious impact on the functionality of these services, with 81% of CR programs reporting interruption or termination of their care activities, in addition to another 14% of services that reduced the number of spots that were available up to that time.^8^ There is no scientific information regarding the return of these services to date; however, informally, it is estimated that the service capacity has not yet returned to the pre-pandemic level. Furthermore, the CR services that resumed their activities began to receive a new flow of post-COVID-19 patients, which may have further reduced the availability of spots for patients with chronic or acute heart diseases after hospital discharge, making the “catastrophic” scenario of CR in Brazil even worse.

## What is the current scenario of cardiac rehabilitation in Portugal?

In recent years, CR has shown progressive improvement, both in number and in quality. However, it is known that it is still far from ideal. In this scenario, the Study Group on Exercise Physiology and Cardiac Rehabilitation of the Portuguese Society of Cardiology has applied recurring questionnaires at CR centers to assess the evolution of CR in Portugal.^9 - 13^ In the latest survey, dated 2019,^13^ 25 centers with CR programs were registered (a 33% increase in relation to 2014),^12^ which is thus considered in accordance with national and European standards.^14 , 15^ Concerning these centers, 11 are located in the North Region, 1 in the Central Region, 12 in the Greater Lisbon Region, and 1 in the South Region. This concentration of centers is related to larger cities and the coastal region of the country, which reflects the great heterogeneity, with a manifest lack of rehabilitation centers in rural regions, small towns, and villages, as well as in the interior, which goes against the recommendations of the World Health Organization for equal access to health.^16^ If we consider that approximately 10,000 people in Portugal are discharged from the hospital annually due to myocardial infarction, and, if they were evenly distributed throughout the national territory (which is not the case), theoretically, each rehabilitation center would have to rehabilitate 400 patients, only in relation to this disease (excluding other causes of cardiovascular disease). Assuming that phase 2 programs last 3 months, how many and which centers would be able to rehabilitate 100 patients at a time, adding to that the numbers of patients with other diseases? And how to achieve phase 3 in the long term?

It is not only in the geographical distribution of centers throughout the territory of Portugal that this great heterogeneity is observed, but also in the types of programs instituted, with varying design and duration. Most phase 2 programs have a weekly frequency of 2 to 3 exercise sessions (ranging from 12 to 36 weeks). In turn, phase 3 programs have a frequency of 1 to 3 sessions per week, with a very long-term option in some cases. All centers have multidisciplinary teams, always including a cardiologist, who often coordinates the programs. In addition to exercise, the programs always included nutritional counseling, most with risk factor control, some with smoking cessation and psychological intervention. In other words, this demands the preparation of teams capable of intervening through the various components of CR, which is already a reality in some hospitals and universities, as is the case with the Masters in Cardiovascular Rehabilitation at the Faculty of Medicine of the University of Lisbon,^17^ a postgraduate program geared toward different specialists. In fact, the availability of qualified professionals may even be a key factor in the emergence of new CR programs.

Another interesting piece of data is that, in 2019,^13^ a 13% increase was found in the number of patients who were referred for CR, when compared to the previous questionnaire from 2014,^12^ especially in the public health system. However, despite the increase in the number of rehabilitated patients, only 9.3% of patients with acute coronary syndrome (ACS), which is the most frequent cause of rehabilitation, were actually rehabilitated. Therefore, if we consider that 100% of these patients should participate in CR programs to improve their quality of life and reduce reinfarction and mortality,^4 , 18^ we are facing a truly suboptimal scenario. In turn, HF of different etiologies was the second most frequent indication for phases 2 and 3 (with 1.8% increase in relation to the previous 2014 questionnaire),^12^ including rehabilitation after implantation of cardiac devices (a 3.1% increase). Certainly, the referral to CR programs of patients after ACS or patients with diagnosis of HF needs to be improved, in addition to the availability and training of the centers.

The causes for non-adherence of patients to CR programs in Portugal, as in other countries, are transportation difficulties, distance to the rehabilitation center, financial problems, lack of family support, lack of motivation, lack of knowledge regarding the benefits and risks of exercise, and labor problems. Further posing difficulties, in the past 2 years, the COVID-19 pandemic has been added to the causes of non-adherence, leading some centers to develop distance programs to attempt to minimize the obstacle of social isolation.

Finally, in spite of significant improvements, CR in Portugal is still far from what would be desirable, both in terms of referral and in terms of better distribution of programs throughout the country.

## Conclusion

The scenario of CR in Brazil and Portugal is still inadequate, and it requires great investment, especially if we consider that this secondary prevention intervention is essential and can save lives. In this context, for effective implementation we need the following:

To promote adequate dissemination of CR through the media and to encourage health policies through scientific societies and health professionals, both individually and institutionally;

To increase referral by means of prevention education for health professionals and the creation of a patient referral system, with indication as early as hospital discharge;

To combat the causes of non-adherence of patients to CR programs, such as: a) transportation difficulties and distances to rehabilitation centers; b) financial problems and lack of family support; c) lack of motivation and lack of knowledge regarding the benefits and risks of exercise; d) work problems.

Finally, it is clear to us that if all the agents potentially involved in the process of implementing rehabilitation programs in both countries (governments, health agents, civil society, and others) invest a fair share, the returns will far exceed the principal. In other words, by practicing medicine based on the best evidence, with a relatively low cost, the result can be a substantial gain in lives, reduced suffering, and even a significant decrease in population health expenditures.
